# Effects of cold treatments on fitness and mode of reproduction in the diploid and polyploid alpine plant *Ranunculus kuepferi* (Ranunculaceae)

**DOI:** 10.1093/aob/mcy017

**Published:** 2018-02-15

**Authors:** Simone Klatt, Christoph C F Schinkel, Bernhard Kirchheimer, Stefan Dullinger, Elvira Hörandl

**Affiliations:** 1 Department of Systematics, Biodiversity and Evolution of Plants, University of Goettingen, Goettingen, Germany; 2 Department of Botany and Biodiversity Research, University of Vienna, Vienna, Austria

**Keywords:** Alpine plants, apomixis, cold stress, fitness, FCSS, polyploidization, Ranunculaceae, *Ranunculus*, *kuepferi*, reproduction mode

## Abstract

**Background and Aims:**

Alpine plants grow in harsh environments and are thought to face occasional frost during the sensitive reproductive phase. Apomixis (asexual reproduction via seed) can be advantageous when sexual reproduction is disturbed by cold stress. Apomictic polyploids tend to grow in colder climates than their sexual diploid relatives. Whether cold temperatures actually induce apomixis was unknown to date.

**Methods:**

We tested experimentally in climate cabinets for effects of low temperatures and repeated frost on phenology, fitness and mode of reproduction in diploid and tetraploid cytotypes of the alpine species *Ranunculus kuepferi*. The reproduction mode was determined via flow cytometric seed screening (FCSS).

**Key Results:**

Diploids produced the first flowers earlier than the tetraploids in all treatments. Cold treatments significantly reduced the fitness of both cytotypes regarding seed set, and increased the frequency of apomictic seed formation in diploids, but not in tetraploids. Over consecutive years, the degree of facultative apomixis showed individual phenotypic plasticity.

**Conclusions:**

Cold stress is correlated to expression of apomixis in warm-adapted, diploid *R. kuepferi*, while temperature-tolerant tetraploids just maintain facultative apomixis as a possible adaptation to colder climates. However, expression of apomixis may not depend on polyploidy, but rather on failure of the sexual pathway.

## INTRODUCTION

Temperature stress is thought to affect many developmental processes in the life cycle of flowering plants (Hedhly, 2011). Plant reproduction in alpine habitats is often affected not only by long periods of snow cover and a resulting short growth period, but also by cold spells with nocturnal frost in spring or summer during development of leaves, shoots or reproductive organs (Körner, 2003; Nagy and Grabherr, 2009). Despite a general adaptation to the cold climate in herbaceous plants of the alpine belt, the frost resistance of reproductive tissue in the bud stage, during anthesis and shortly after anthesis is relatively low compared with vegetative organs (Ladinig *et al.*, 2013; Kuprian *et al.*, 2014). Moreover, freezing can negatively influence, for example, flowering time or cause defects in male or female gametes resulting in poor seed set (Thakur *et al.*, 2010; Zinn *et al.*, 2010; Hedhly, 2011; De Storme and Geelen, 2014). As a corollary, sexual plant reproduction might increasingly fail at higher altitudes (Neuner *et al.*, 2013) as has been shown, for example, for *Ranunculus acris* populations in alpine areas of Norway (Totland, 1997). Developmental plasticity such as overinvestment in ovule production (Wagner *et al.*, 2016), flexible timing of flowering (Nicotra *et al.*, 2015) or a change of reproduction mode from sexuality to asexuality (Schinkel *et al*., 2016, 2017) are potential strategies that facilitate plant propagation in cold environments.

Low temperatures may have a direct effect on polyploidization and mode of reproduction (Ramsey and Schemske, 1998). A spontaneous production and fusion of diploid (unreduced) male and female gametes is considered the major factor for the development of autopolyploids (Bretagnolle and Thompson, 1995). New polyploid cytotypes often arose in regions with changing environmental conditions such as mountainous, previously glaciated areas (Stebbins, 1971; Bretagnolle and Thompson, 1995). Environmental factors such as low temperatures are discussed as a trigger for unreduced gamete formation (Felber, 1991; Bretagnolle and Thompson, 1995; Mason *et al.*, 2011). Cold effects on male meiosis are disturbances of spindle and cell wall formation, resulting in irregular meiotic cell division with subsequent production of diploid and polyploid male gametes (pollen) (De Storme *et al.*, 2012; De Storme and Geelen, 2014; Mirzaghaderi and Hörandl, 2016, and references therein). Less is known about the impact of cold stress on female sporogenesis and gamete formation. A disruption of female meiosis after chilling stress (Thakur *et al.*, 2010) could produce unreduced female gametes after so-called restitutional meiosis (diplospory) or can trigger the development of apomeiotic (aposporous) cells into unreduced embryo sacs. Accordingly, we hypothesize that the apomictic programme might be started as an alternative to the sexual reproductive pathway in the wake of cold stress.

The production of unreduced female gametes from unreduced embryo sacs is the first step of gametophytic apomictic reproduction. Apomixis means asexual reproduction via seeds (Nogler, 1984) and is known to exist in >290 angiosperm genera (Hojsgaard *et al.*, 2014*a*). During gametophytic apomixis, an unreduced embryo sac is formed from a somatic cell of the nucellus tissue (apospory) or from an unreduced megaspore resulting from restitutional meiosis of the megaspore mother cell (diplospory) (Asker and Jerling, 1992; Koltunow and Grossniklaus, 2003). Functional apomixis comprises three processes: circumvention of meiosis during unreduced embryo sac formation (apomeiosis), the parthenogenetic development of the egg cell into an embryo and the formation of endosperm tissue after fertilization of the two polar nuclei with a sperm cell (pseudogamy) or without male contribution (autonomous endosperm) (Koltunow and Grossniklaus, 2003; Hand and Koltunow, 2014). Most natural apomicts are facultative, which means that an individual plant is able to produce sexual and asexual seeds in varying frequencies (Hand and Koltunow, 2014).

Spatial and temporal changes in the expression of genes related to the sexual pathway and epigenetic mechanisms can initiate apomixis processes (Grimanelli, 2012; Hand and Koltunow, 2014; Shah *et al.*, 2016). However, the genetic control and epigenetic regulation of apomixis, and environmental influence on expression of the trait are still not completely understood. Facultatively apomictic plants were found to enhance the frequency of sexual ovules under light stress conditions (Quarin, 1986; Klatt *et al.*, 2016) or under cultivation at elevated temperature (Šarhanová *et al.*, 2012). The effects of cold stress are unknown, but we would conversely expect an increase in the proportions of apomictic seeds. However, for most facultative apomicts, it is even unknown whether the mode of reproduction remains stable in the same individuals over consecutive years.

Apomicts are not restricted to cold environments (Hörandl *et al.*, 2011), but polyploid apomicts are often more widespread at higher altitudes and latitudes than their close diploid sexual relatives (‘geographical parthenogenesis’; Vandel, 1928; Bierzychudek, 1985; Hörandl, 2006). Traditional hypotheses explain this phenomenon with better colonization abilities and/or ecological adaptations of polyploids (Hörandl, 2006; Burnier *et al.*, 2009; Cosendai *et al.*, 2013; Kirchheimer *et al.*, 2016). Here we want to test the hypothesis that cold climates have a direct, physiological effect on mode of reproduction. Even if the capability of producing asexual seeds is heritable, variable frequencies of sexual and asexual seeds in facultatively apomictic plants indicate that the actual expression might be influenced by abiotic environmental conditions (Quarin, 1986; Šarhanová *et al.*, 2012; Klatt *et al.*, 2016). We suppose that diploid and polyploid plants differ in their stress response system (Comai, 2005; Ramsey and Ramsey, 2016; Shah *et al.*, 2016; Schoenfelder and Fox, 2015). Whether stress alters the reproduction mode of plants towards asexuality might depend on the species and its sensitivity to changes in abiotic factors (Hörandl and Hadacek, 2013). Moreover, the timing, combination and intensity of the stress parameters may play an important role (e.g. Suzuki *et al.*, 2014).

Our model system is *Ranunculus kuepferi* Greuter & Burdet, a perennial herb distributed mainly across the European Alps. The white-flowered buttercup *R. kuepferi* occurs with several cytotypes in alpine grassland at altitudes between 1300 and 2800 m (Kirchheimer *et al.*, 2016). Diploid populations (2*n* = 16) are restricted to the south-western Alps whereas tetraploids (2*n* = 32) cover a wide range of the European Alps and are mainly found in previously glaciated areas (Küpfer, 1974; Cosendai and Hörandl, 2010). Tetraploids occur at higher elevations in the European Alps, and exhibit a pronounced niche shift towards colder temperatures (Kirchheimer *et al.*, 2016; Schinkel *et al.*, 2016). Previous studies revealed that tetraploid cytotypes arose several times from diploid progenitors by autopolyploidization events (Cosendai *et al.*, 2011). Tri-, penta- and hexaploid cytotypes appear in minor frequencies in the contact zones of diploid and tetraploid populations (Küpfer, 1974; Burnier *et al.*, 2009; Cosendai and Hörandl, 2010; Cosendai *et al.*, 2013; Schinkel *et al.*, 2016). Under natural conditions, diploid plants of *R. kuepferi* are predominantly sexual, but a few apomictic seeds appeared in three populations (Schinkel *et al.*, 2016). Tetraploids turned out to be facultatively sexual/apomictic (aposporous; Burnier *et al.*, 2009) and produce sexual and asexual seeds in varying proportions (Schinkel *et al.*, 2016). We observed a positive correlation of low temperature and asexual reproduction in wild tetraploid populations under natural conditions (Schinkel *et al.*, 2016).

Here we use experimental treatments to test specifically whether cold temperatures and frost trigger apomixis. Using flow cytometric seed screening (FCSS), we indirectly observed the mode of seed formation via ploidy determination of the embryo and endosperm tissue. We aim to answer the following questions. (i) Do cold treatments with repeated frost affect reproductive development and fitness of diploid and polyploid plants differently? (ii) Do cold treatments and frost during the reproductive phase change the mode of reproduction of sexual diploids and facultatively sexual/apomictic tetraploids? (iii) How stable is the mode of reproduction of the same individual in consecutive years? (iv) Do cold treatments promote pathways to polyploidization of diploid and tetraploid cytotypes via ploidy shifts in the embryo?

## MATERIALS AND METHODS

### Plant material and experimental set-up

Plants of the diploid and the tetraploid cytotype of *Ranunculus kuepferi* were collected during the growing seasons of 2013 and 2014 in the European Alps at 102 sampling sites encompassing the whole alpine distribution range of the species (for detailed information on sampling design and geographical range, see Kirchheimer *et al.*, 2016). The plants were transferred to the Old Botanical Garden of Göttingen University (Germany) and were re-potted in garden soil. Since *R. kuepferi* is perennial, all experiments could be performed on the same individuals over 2 years. Plants overwintered outdoors in the garden. The ploidy level of individuals was determined via flow cytometry of silica gel-dried leaf material (Schinkel *et al.*, 2016). When they started to foliate in early spring (mid March 2014), sub-sets of diploid and tetraploid individuals were exposed to different temperature conditions in two climate cabinets MC1000E (Snijders Scientific, Tilburg, The Netherlands) and outdoors in the Botanical Garden of the University Göttingen, respectively. Temperature and light settings are detailed in Table 1. The cold treatment simulated temperature conditions in harsh alpine environments especially typical for the habitats of the tetraploid *R. kuepferi* cytotype (Schinkel *et al.*, 2016). We applied a repeated moderate frost treatment as frost injury in reproductive shoots could result in full fruit loss even in cold-adapted high-mountain plants (Ladinig *et al.*, 2013). Tested individuals originated from 64 populations representative for the whole distribution area of the species in the European Alps (see Supplementary Data Table S1 for the geographical provenance of populations). Voucher specimens were deposited after the experiment in the herbarium of the University of Göttingen (GOET).

**Table 1.** Number of investigated diploid and tetraploid *Ranunculus kuepferi* plants and growth conditions in temperature treatments in two experimental years (2014 and 2015)

Plant ploidy	Cold treatment		Warm treatment		Outdoor treatment*	
	Diploid	Tetraploid	Diploid	Tetraploid	Diploid	Tetraploid
No. of plants in 2014	51	56	52	53	53	53
No. of plants in 2015	100	102	92	102	–	–
	Conditions during plant growth and seed maturation					
Temperature during the light/dark period (°C)	+7/–1 (three nights)^†^ +7/+2 (four nights)		+15/+10		Central European lowland temperatures Mid March–Mid June^‡^	
Length of light/dark period (h)	16/8^§^ (10 full light, and 3 + 3 twilight)				Natural daylength, days becoming longer during the experiment	
Light intensity (µmol m^–2^ s^–1^, PAR)	740 in maximum^¶^				Natural day light	

^*^Old Botanical Garden, University of Göttingen, Germany, 51.538° (N), 9.939° (E), 150 m asl.

^†^Three successive nights each week until seed harvest.

^‡^Minimum +0.6 °C (26 March), maximum +38.6 °C (9 June); recorded with iButton™ (Maxim Integrated Products, Inc., Sunnyvale, CA, USA) at ground level; for temperature profiles, see Supplementary Data Fig. S1

^§^According to Ladinig *et al.* (2013).

^¶^Measured with a Quantum light meter (Spectrum Technologies Inc., Aurora, IL, USA) during the full light period (100 % intensity) at the level of early leaf tips and first (often stalkless) buds. Plants were rotated weekly in the cabinet to avoid effects of light and temperature gradients.

### Monitoring of flowering

**Fig. 1.**
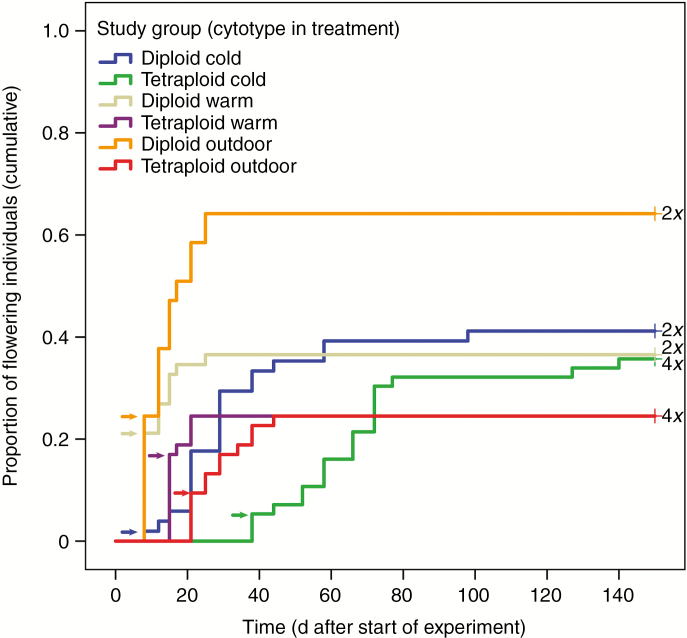
Cumulative flowering curves of diploid and tetraploid *Ranunculus kuepferi* plants under controlled cold and warm conditions in climate chambers and under outdoor conditions (Old Botanical Garden, University of Göttingen, Germany), experimental year 2014. Arrows mark the beginning of flowering for each group.

The influence of the treatment on plant development was observed by monitoring the flowering success of all individuals. In 2014 the developmental stage of the plants (number of buds, flowers and seed heads) was recorded at least weekly until plants started fruiting. Time to flowering of the study groups (treatments and cytotypes) was compared via survival analysis, and observations were plotted as a percentage of flowering individuals (flowering rate) against the time after the start of the experiment (start of plant development after winter) (Fig. 1; Kaplan–Meier curves). Plants were recorded as being in the flowering stage when buds were open and carpels and stamens visible.

### Reproductive fitness/seed set

We tested whether cold temperatures and frost had a significant influence on the reproductive fitness of diploid and tetraploid *R. kuepferi* plants by analysing the seed set (percentage of well-developed seeds as a proportion of all seeds of a flower) of all individuals in the controlled cold and warm treatment in two experimental years, and in the outdoor treatment in the first year. Flowers of the diploid cytotype of *R. kuepferi* have fewer carpels, more well-developed stamens and are known to be self-sterile, whereas the tetraploid cytotype often shows a 3-fold higher number of carpels, only a few stamens, and is (partly) self-fertile. Pollen in apomictic tetraploids is needed for fertilization of the polar nuclei for proper endosperm formation, while the egg cell remains unfertilized (pseudogamy) (Küpfer, 1974; Huber, 1988; Burnier *et al.*, 2009; Cosendai *et al.*, 2013). In our experiments, all diploid and tetraploid individuals were pollinated manually at least twice with pollen from plants of the same ploidy level in the same treatment. Cross-pollination between diploid and tetraploid plants was avoided by covering fertile flowers with small porous plastic bags. After pollination, flowers were bagged with porous plastic bags sealed with tape to avoid seed loss until harvesting of mature fruits. Seed set was assessed as the percentage of well-developed achenes among all achenes after Schinkel *et al.* (2016). Well-developed seeds were stored in Eppendorf tubes at 4 °C until flow cytometric measurements.

### Ploidy determination in single seeds (FCSS)

We tested whether temperature had an effect on the proportions of sexual vs. asexual seed formation of *R. kuepferi* grown under cold (with frost nights) and warm conditions in the cabinets using FCSS.

The ratio of embryo:endosperm ploidies differs in sexual and asexual seeds due to reduced vs. unreduced embryo sac formation (Matzk *et al.*, 2000; Table 2). The ploidy levels of the embryo and the endosperm in up to ten seeds of each individual of all temperature treatments in 2014 and 2015 were measured and the reproductive pathway was calculated for each seed following a slightly modified FCSS procedure originally described by Schinkel *et al.* (2016). Single seeds were ground by two steel beads (Qiagen, Hilden, Germany) (Ø 4 mm) in a 2 mL Eppendorf tube with a Tissue Lyzer II (Qiagen; stroke rate 30 Hz, time 7 s). Nuclei were isolated and stained in two steps using Otto buffers (Otto, 1990; Doležel and Bartoš, 2005; Doležel *et al.*, 2007). In the first step, the ground seed material was mixed with 200 µL of Otto I buffer for 30 s to extract nuclei from the cells. After filtration of this mixture (30 µm mesh, CellTrics^®^ Partec GmbH, Münster, Germany) into plastic tubes (3.5 mL, 55 × 12 mm, Sarstedt, Nümbrecht, Germany), 800 µL of Otto II buffer [staining solution with 4’,6-diamidino-2-phenylindole (DAPI) with a concentration of 300 µg mL^–1^] was added to the filtrate and the solution was measured directly in a flow cytometer (CyFlow Space, Partec GmbH, Münster, Germany) in the blue fluorescence channel (UV LED, wavelength 365 nm). The DNA content (ploidy) of the nuclei is proportional to the detected fluorescence intensity. A diploid *R. kuepferi* plant was used as the external reference to adjust the gain standard of the UV lamp (Schinkel *et al.*, 2016) and the parameters were kept for all measurements. Gaussian means of the peaks were analysed with the software FloMax version 2.81 (Quantum Analysis GmbH, Münster, Germany), and peak indices (mean peak value of the embryo compared with mean peak value of the endosperm) were calculated (Microsoft Excel 2007).

**Table 2.** Reproduction modes observed for diploid and tetraploid *Ranunculus kuepferi* in temperature experiments (2014 and 2015 combined)

Reproduction mode	Genome contribution to embryo/endosperm	Embryo:endosperm	Peak index	Number of observations (seeds, ssFCSS) in two experimental years			
Diploid plants	Egg cell + sperm nucleus/fused polar nuclei + sperm nucleus (nuclei)			Cold	Warm	Outdoor*	Total
Sexual	1Cx(m) + 1Cx(p)/2Cx(m) + 1Cx(p)	2:3	1.5	186	588	318	1092
Apomictic	2Cx(m) + 0Cx(p)/4Cx(m) + 0Cx(p)^†^	2:4	2.0	3	2	3	8
	2Cx(m) + 0Cx(p)/4Cx(m) + 2Cx(p)^‡^	2:6	3.0	4	2	0	6
B_III_ hybrid	2Cx(m) + 1Cx(p)/4Cx(m) + 1Cx(p)	3:5	1.67	0	2	0	2
	2Cx(m)+1Cx(p)/[4Cx(m) + 1Cx(p)] × 2^§^	3:10	3.3	0	2	0	2
Tetraploid plants							
Sexual	2Cx(m) + 2Cx(p)/4Cx(m) + 2Cx(p)	4:6	1.5	6	24	7	37
Apomictic	4Cx(m) + 0Cx(p)/8Cx(m) + 0Cx(p)^†^	4:8	2.0	1	3	0	4
	4Cx(m) + 0Cx(p)/8Cx(m) + 2Cx(p)^¶^	4:10	2.5	74	261	49	384
	4Cx(m) + 0Cx(p)/8Cx(m) + 4Cx(p)^‡^	4:12	3.0	21	61	16	98
	4Cx(m) + 0Cx(p)/8Cx(m) + 6Cx(p)**	4:14	3.5	6	10	7	23
	4Cx(m) + 0Cx(p)/8Cx(m) + 8Cx(p)^††^	4:16	4.0	1	3	3	7
B_III_ hybrid	4Cx(m) + 2Cx(p)/8Cx(m) + 2Cx(p)	6:10	1.67	0	3	1	4

Cx reflects ploidy based on DNA content; m, maternal genome contribution; p, paternal genome contribution.

*Outdoor group in 2014 only, plants in the Old Botanical Garden, University of Göttingen, Germany.

^†^Autonomous endosperm.

^‡^Pseudogamous endosperm, fertilized with one unreduced or two reduced sperm nuclei.

^§^Endosperm endopolyploidy.

^¶^Pseudogamous endosperm, fertilized with one reduced sperm nucleus.

**Pseudogamous endosperm, with one reduced and one unreduced sperm nucleus (or two reduced nuclei with approx. 3Cx or trinucleate endosperm; see Schinkel *et al.*, 2016).

^††^Pseudogamous endosperm, with two unreduced sperm nuclei or endosperm endopolyploidization, see Schinkel *et al.* (2016).

Mean peak positions of the embryo and the endosperm DNA content reveal the ploidy levels of both tissues and allow for the interpretation of their formation (Table 2). Ploidy level based on DNA content is given as Cx value following the terminology of Greilhuber *et al.* (2005). Seeds with peak indices <1.65 were categorized as sexually formed seeds. Typically the peak index of sexual seeds is around 1.5 (embryo:endosperm = 2Cx:3Cx and 4Cx:6Cx for diploid and tetraploid plants, respectively). Peak indices above the threshold of 1.65 indicate asexually formed seeds with an unreduced egg cell developing parthenogenetically into an embryo (2Cx), and the two unreduced polar nuclei (4Cx) fertilized by either reduced (1Cx) or unreduced (2Cx) male sperm nuclei (pseudogamous endosperm).The resulting genome contributions of embryo:endosperm and the respective peak indices (endosperm/embryo) are detailed in Table 2 for all developmental pathways observed in our study. For examples of apomictic seed formation pathways analysed by FCSS data, see, for example, Matzk *et al*. (2000, 2001); Cosendai and Hörandl (2010); Šarhanová *et al*. (2012); Hojsgaard *et al.* (2014*b*); Klatt *et al.* (2016); and Schinkel *et al.* (2016). Representative peak indices for full apomictic seeds are 2.5, 3.0, 3.5 and 4.0. Few measurements resulted in a ratio of 2.0 (peak indices between 1.85 and 2.15) referring to autonomous endosperm formation without fertilization of the polar nuclei (Table 2). In addition to full sexual and full apomictic seed formation, where the ploidy of the embryo is the same as that of the mother plant, the FCSS method enables detection of seeds with ploidy shift of the embryo. Here we detected 3Cx for diploids and 6Cx for tetraploids, due to fertilization of an unreduced egg cell by a male sperm nucleus; the endosperm ploidies are 5Cx and 10Cx, respectively (Table 2). These so-called B_III_ hybrids (Nogler, 1984) were rare, but we included them as a third category in our statistics as they represent cases of partial apomixis (i.e. apomeiosis only) and possible pathways to polyploidization. Representative flow histograms are shown in Supplementary Data Fig. S2. The percentages of sexual, asexual and B_III_ seeds were calculated for each individual and results were pooled for ploidy levels to evaluate the influence of temperature treatments on reproduction mode. From 1705 FCSS measurements, 1667 cases were clearly interpretable (Table 2), but 38 cases were excluded from further statistical analyses because of potential irregularities during embryo and endosperm development (Supplementary Data Table S2).

### Statistical analysis

We used Kaplan–Meier curves to visualize the duration of time from the start of the experiment to the time when individuals started flowering in both cytotypes (IBM SPSS Statistics 24). The Kaplan–Meier estimator is a non-parametric statistic used to estimate waiting times until a specified event from observations (taken at intervals, weekly in our case) in the form of so-called survival curves (Kaplan and Meier, 1958). We subsequently used log rank tests (= Mantel–Cox tests) to test for the influence of cytotype and treatment on these survival curves, i.e. whether the time until flowering differed among the cytotypes and among treatments (IBM SPSS Statistics 24). Potential predispositions regarding flowering start due to climatic conditions at the origin of each plant individual were tested via linear regressions including time until flowering and altitude of origin. A general linear model with post-hoc Bonferroni test was used to compare the flowering rate of the study groups by day 150 (last flowers observed) (IBM SPSS Statistics 24). To test for the influence of the temperature treatment on seed set and on reproduction mode of diploids and tetraploids, we used non-parametric Kruskal–Wallis and Mann–Whitney U-tests. Percentage values were arcsine transformed prior to statistical analysis. Microsoft Excel 2007 and IBM SPSS Statistics 24 were used for calculations of descriptive statistics and graphical presentations. Boxplots were plotted with untransformed percentage values and show the 25th and 75th percentile ranges as a box and the median as a black line; circles are outliers, asterisks are extreme values.

## RESULTS

### Temperature effects on flower formation

Flowering started in diploids on the eighth day in all treatments, while tetraploids started to flower later (on the 15th, 21st and 38th day in the warm, outdoor and cold treatments, respectively (see arrows indicating starting days in Fig. 1). Survival analysis (including all individuals of a group) revealed that plants in the different treatments differed significantly in the time needed until flowering (*χ*^2^ = 39.889, *P* = 0.000). In particular, the diploid plants grown outdoors needed a shorter period until flowering than the diploids grown under controlled cold (*χ*^2^ = 11.290, *P* = 0.001) and warm (*χ*^2^ = 6.570, *P* = 0.010) conditions. Flowering rate (proportion of flowering plants at day 150) in outdoor-grown diploid plants was highest, and differed significantly from that of the warm-treated diploids (*P* = 0.043), but not from that of the cold-treated diploid plants (*P* = 0.2, Bonferroni test). For the tetraploids, times until flowering and flowering rates at day 150 did not differ significantly between the three treatments (*P* > 0.05).

A highly significant positive correlation of time until flowering and altitude of origin was observed for the outdoor-grown diploid plants (*R*^2^ = 0.505, *P* = 0.000) as well as a significant positive correlation for the other diploid study groups (cold treatment, *R*^2^ = 0.240, *P* = 0.024; warm treatment, *R*^2^ = 0.260, *P* = 0.026). For the tetraploid cytotype, no significant correlations between time until flowering and altitude of origin were found (*P* > 0.05).

### Seed set according to different temperature and frost treatments

**Fig. 2.**
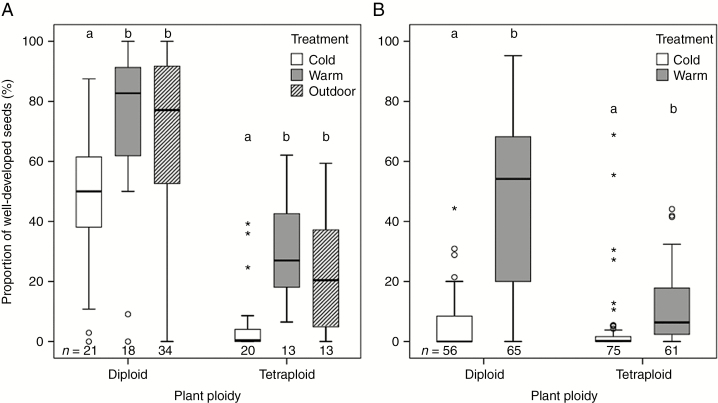
Influence of temperature on reproductive fitness (seed set) in diploid and tetraploid *Ranunculus kuepferi* plants (A) in 2014 (cold, warm and outdoor group) and (B) in 2015 (cold and warm group). *n* = number of individuals. For test statistics, see Supplementary Data Table S3.

The temperature during growth and the reproductive phase had a significant influence on the production of seeds in both cytotypes of *R. kuepferi*. Seed set (of flowering individuals) was significantly reduced in the cold treatment in both experimental years, while warm and outdoor conditions had similar positive effects on seed production (*P* < 0.01, Fig. 2A, B; Supplementary Data Table S3). The mean seed set of diploids was at all times higher than the seed set of tetraploids of the same temperature treatment (Supplementary Data Table S3). Diploid individuals in the cold treatments in both years produced significantly less well-developed seeds compared with controlled warm- and outdoor-grown diploids (first year, *P* < 0.01, Fig. 2A; second year, *P* < 0.001, Fig. 2B; all data are given in Supplementary Data Table S3). Tetraploid individuals were also significantly influenced by cold treatments. Their seed set was reduced compared with warm and outdoors (first year, *P* < 0.01, Fig. 2A; second year *P* < 0.001, Fig. 2B; Supplementary Data Table S3). In the second year, the negative influence of low temperature on seed set was higher for diploids than for tetraploids (Fig. 2B; Supplementary Data Table S3).

### Temperature effects on mode of reproduction

**Fig. 3.**
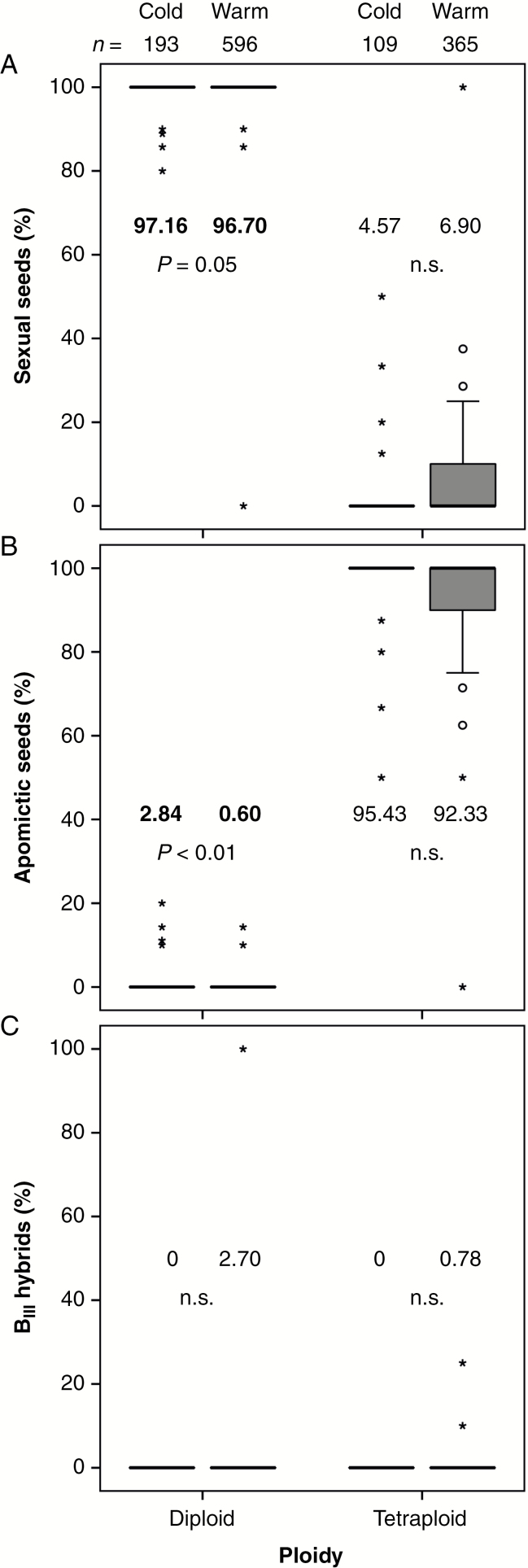
Influence of temperature treatments on the mode of reproduction in diploid and tetraploid *Ranunculus kuepferi* plants (pooled data of two experimental years). Boxplots show the percentages of (A) sexual seeds, (B) apomictic seeds and (C) B_III_ hybrids (partially asexual seeds) produced by plants in the cold and warm treatment. Mean values and statistical significance are given in the figure. *n* = number of seeds in the study group. For test statistics, see Supplementary Data Table S4a.

To detect temperature effects on the mode of reproduction, the FCSS data of both experimental years were pooled for the cold and warm treatment, respectively (Fig. 3; Table 2). The results for the outdoor treatment were excluded as they did not differ significantly from those of the warm treatment (Supplementary Data Fig. S3; Table S4). As expected, diploid plants developed predominantly sexual seeds, while tetraploid plants produced predominantly apomictic seeds, irrespective of temperature treatment (Fig. 3; Supplementary Data Table S4). Interestingly, both cytotypes in our experiment turned out to be facultatively apomictic and showed flexibility in reproduction mode. Cold-treated diploid plants had a small but significantly higher mean proportion of apomictic seeds [2.84 ± 5.67 % (s.d.)] than warm-treated diploids (0.60 ± 2.56 %, *P* < 0.01). Tetraploids produced about the same proportions of apomictic seeds in the cold treatment (mean 95.43 %) as in the warm treatment (mean 92.33 %, difference not significant). Partial apomixis with ploidy shifts in the embryo occurred in the form of B_III_ hybrids in warm-treated diploids and tetraploids, but not in the cold treatment (Fig. 3; Supplementary Data Table S4). The production of sexual seeds is positively correlated with the percentage of well-developed seeds (*r* = 0.659, *P* < 0.01).

### Reproductive behaviour of wild *R. kuepferi* in natural environments and in experiments

To test for individual reproductive flexibility, the results of our repeated controlled temperature experiments were compared with data of the same individuals from their natural habitat in the Alps (Schinkel *et al.*, 2016). The results could shed light on the question of whether changes from obligate sexual to facultative asexual reproduction in diploids and the reverse for tetraploids are clearly driven by temperature conditions, are genetically fixed or are spontaneous events in predisposed plants. Despite repetition of our experiments with the same sub-set of plants, reliable annual reproduction data for single individuals were not easy to obtain, as many individuals only flowered in the first or the second year, and not in both. Moreover, some plants produced fewer than five evaluable seeds, the minimum threshold we set for comparisons. Therefore, the overview of the reproductive flexibility of *R. kuepferi* individuals given in Table 3 contains only 19 plants with evaluable seed numbers in 2 or 3 years during our investigations (Schinkel *et al.*, 2016; this study). Among diploid sexual individuals, one individual changed from sexual reproduction in the wild to apomictic reproduction during the cold treatment (Table 3). Among the 18 tetraploids, one individual (5.6 % of individuals) changed from pure apomictic to mixed sexual/apomictic reproduction, while one plant (5.6 %) changed from mixed to pure apomictic seed production during the cold treatment. In tetraploid individuals that were transferred from natural sites to warm treatments, shifts towards apomixis occurred in eight plants (44.4 %), while shifts to sex occurred in six plants (33.3 %) (Table 3). Shifts in B_III_ seed formation in the direction of less partial asexuality occurred in two plants (11.1 %) under warm conditions (Table 3).

**Table 3.** Reproductive flexibility [production of sexual, apomictic, partially asexual (B_III_) seeds] of wild diploid and tetraploid *Ranunculus kuepferi* individuals in their natural environment and in temperature experiments

Altitude in m asl/region in the Alps*	Individual	Plant ploidy	Natural conditions (Alps)^†^	Cold/frost experiment^‡^		Warm experiment (includes outdoor lowland 2014)^‡,§^	
			2013/2014	2014	2015	2014	2015
Diploid sexual individuals producing apomictic seeds							
Observations in three successive years							
1925/Provence-Alpes-Côte d’Azur	24-3-2	2*x*	0 apo/5 sex	–	**1 apo**/4 sex	0 apo/10 sex	–
Tetraploid facultative asexual individuals changing frequencies							
Observations in three successive years							
2357/Provence-Alpes-Côte d’Azur	17-2-1	4*x*	1 sex/4 apo	0 sex/**7 apo**	0 sex/**7 apo**	–	–
2117/Trentino Alto Adige	58-1-3	4*x*	2 sex/3 apo	–	–	0 sex/**7 apo**	0 sex/6 apo
2211/Graubunden	47-1-2	4*x*	1 sex/4 apo	–	–	0 sex/**10 apo**	**2 sex**/5 apo
2211/Graubunden	47-1-3	4*x*	1 sex/4 apo	–	–	0 sex/**10 apo**	0 sex/9 apo
2300/Provence-Alpes-Côte d’Azur	96-2-3	4*x*	3 sex/2 apo	–	–	1 sex/**9 apo**	**1 sex**/4 apo
2300/Provence-Alpes-Côte d’Azur	96-3-2	4*x*	0 sex/5 apo	–	–	**4 sex**/4 apo	3 sex/**5 apo**
2357/Provence-Alpes-Côte d’Azur	17-3-1	4*x*	0 sex/5 apo	–	–	**2 sex**/7 apo	2 sex/6 apo
Observations in 2 years							
2152/Rhônes-Alpes	36-2-3	4*x*	0 sex/5 apo	**1 sex/**4 apo	–	–	–
1789/Valais	42-2-3	4*x*	1 sex/4 apo	–	–	0 sex/**9 apo**	(0 sex/3 apo)^¶^
1860/Valais	40-2-2	4*x*	2 sex/3 apo	–	–	0 sex/**10 apo**	(0 sex/4 apo) ^¶^
1860/Valais	40-4-2	4*x*	1 sex/4 apo	–	–	–	0 sex/**8 apo**
2243/Provence-Alpes-Côte d’Azur	111-1-1	4*x*	1 sex/4 apo	–	–	–	0 sex/**7 apo**
2357/Provence-Alpes-Côte d’Azur	17-3-2	4*x*	2 sex/3 apo	–	–	–	0 sex/**5 apo**
2357/Provence-Alpes-Côte d’Azur	17-4-2	4*x*	2 sex/3 apo	–	–	–	1 sex/**8 apo**
2400/Valais	45-4-1	4*x*	0 sex/5 apo	–	–	–	**2 sex**/6 apo
2405/Valais	93-3-3	4*x*	0 sex/5 apo	–	–	–	**3 sex**/5 apo
Tetraploid individuals producing B_III_ hybrids (partial asexuality)**, full apomictic and sexual seeds							
Observations in 2 years							
1860/Valais	40-4-2	4*x*	1 B_III_/4 apo/0 sex	–	–	–	**0 B**_**III**_/8 apo/0 sex
2115/Val d’Aosta	37-3-2	4*x*	1 B_III_/3 apo/1 sex	–	–	**1 B**_**III**_/9 apo/0 sex	–

Plant ploidy and seed reproduction mode were assessed via flow cytometry of leaf material and seeds [*n* = 5 seeds (natural conditions) or up to *n* = 10 seeds (experiments) measured, depending on the actual number of mature seeds per individual] respectively. Only individuals with observations in one of two or three flowering periods, a minimum of five seeds per period and a minimum shift of 20 % frequency are shown. Respective shifts are marked in bold.

Apo, no. of apomictic seeds; sex, no. of sexual seeds; B_III_, no. of seeds with partial sexuality.

*For a complete list of collection sites including co-ordinates, see Schinkel *et al.* (2016)

^†^Seeds collected in 2013 or 2014.

^‡^In a climate cabinet with controlled temperature.

^§^In Göttingen Botanical Garden,

^¶^Not evaluated because there were <5 seeds

**An unreduced egg cell is fertilized with reduced pollen.

–, The individual was not in the experiment or no measurable seeds were produced.

### Temperature effects on polyploidization (female B_III_ hybrids, triploid bridge)

An increase in ploidy level during reproduction was observed in single cases for diploid and tetraploid mother plants in the warm experiments and under natural conditions (Schinkel *et al.*, 2016), but not in the cold/frost treatment (Tables 2 and 3). Partial asexuality (fertilization with the contribution of unreduced female gametes) resulted in 3*x* embryos (diploid mother plants) and in 6*x* embryos (tetraploid mother plants). The ploidy measurements of the embryo and endosperm (FCSS data) shed light on the ploidy of the involved female and male gametes. All detected B_III_ hybrids were derived from the fertilization of an unreduced egg cell with reduced pollen, and are so-called female B_III_ hybrids.

## DISCUSSION

As expected from mid-altitude outcrossers, diploid plants in the outdoor group flowered earlier and with a higher percentage of individuals than all other groups. The relatively high floral display optimizes synchrony of phenology of self-incompatible diploid plants with insect pollinator visits that are thought to be more abundant and active at lower altitudes, especially under warm outdoor conditions. Such conditions resemble the natural habitat of diploid *R. kuepferi* populations in the mid altitudes of the south-western Alps. The correlation of flowering to altitude of origin suggests that diploids partly kept these pre-dispositions in the experiments. From our observations in the wild, we know *R. kuepferi* as an early-flowering species with a short pre-floration period between snowmelt and bloom. Interestingly, the flowering rate of the tetraploid cytotype was affected neither by cold compared with warm treatments, nor by outdoor lowland conditions. Obviously, they did not benefit from warmer temperatures with respect to flower production. Since tetraploids are self-compatible, their reproductive success is largely independent of pollinator visits (Cosendai *et al.*, 2013). This strategy might also be an adaptation to short vegetation periods, reduced or variable pollinator frequencies and pollen availability in cold environments at higher altitudes (Wagner *et al.*, 2016). Altogether, phenology and flower/fruiting rates of tetraploids appear to be quite constant irrespective of altitude of origin and climatic conditions. This broad tolerance may be beneficial under high alpine conditions.

Cold treatments with repeated nocturnal frost reduced seed set in both *R. kuepferi* cytotypes. In this aspect, both cytotypes do not appear well adapted to these conditions although tetraploids were collected from altitudes up to 2700 m asl. A low seed set was also reported for the high-mountain plant *Saxifraga bryoides* (Ladinig and Wagner, 2007). Possible reasons are slow embryo growth and seed loss because of undeveloped embryos under cooler temperatures (Ladinig and Wagner, 2007). Also pollen sterility caused by low-temperature stress at high altitudes is a factor reducing seed set as presumed for 4*x* cytotypes of Himalayan *Ranunculus hirtellus* (Kumar and Singhal, 2011). In our frost-treated diploid plants, the reproductive fitness was drastically reduced after 2 years of cultivation under permanent low temperatures. The lower seed production of diploids in the second experimental year showed that these plants remained frost sensitive and did not adapt to their ‘artificial’ growth conditions. The reasons might be diverse and, apart from disturbances of the male and female sporogenesis or gametogenesis, or hampered embryo development, frost damage of the flower stalks cannot be ruled out. The complete seed abortion of many cold- and frost-treated diploid individuals suggests an all or nothing principle described by Ladinig *et al.* (2013) who observed full fruit loss in alpine herbs when reproductive shoots were injured by frost treatments (between –2 and –14 °C). Polyploid *R. kuepferi* plants do not appear, in general, to be more successful than diploids in our experiments. In both temperature treatments they produced on average less well-developed seeds compared with the diploid plants. This result corresponds well to our observation in wild populations where polyploid *R. kuepferi* plants had lower seed set than diploids. However, only tetraploid plants are capable of producing well-developed seed at the highest elevations in the Alps (2400–2700 m) (Schinkel *et al.*, 2016). Possibly, only tetraploids would be fertile at all under more extreme cold conditions than tested in our experimental set-up.

Our results suggest that cold temperatures and frost could trigger unreduced (female) gamete formation and apomixis in diploids. Under experimental cold conditions with repeated frost events, otherwise sexual diploid *R. kuepferi* plants produced significantly more apomictic seeds compared with warm-treated diploids. Likewise, unreduced egg cells and apomictic seeds were detected in three sexual diploid populations in the natural environment (Schinkel *et al.*, 2016), possibly induced by frost events after snowmelt and during the early reproductive stage of the ovules. However, in facultative tetraploids, the increase of apomictic seed production was not significant after cold and frost treatment. Under natural growth conditions, the mode of reproduction in *R. kuepferi* populations correlated with altitude and corresponding climatic variables, with a tendency for increasing frequencies of apomixis at higher elevations and colder climate (Schinkel *et al.*, 2016). Cold temperatures and frost thus might play a role in stimulating the production of apomictic seeds. Certainly, in natural environments, many other abiotic factors affect growth and photosynthesis, and can influence reproduction directly or indirectly (Körner, 2003). In addition to frost events during summer, plants in high-mountain habitats might be exposed to other stressors such as strong solar radiation, short-term overheating, soil drought or reduced carbon availability due to decreasing CO_2_ partial pressure with increasing altitude (Körner, 2003; Larcher *et al.*, 2010). In general, plants, even though adapted to their natural environment, might be occasionally stressed by a combination of unfavourable conditions such as unusually high temperatures and drought (Ladinig and Wagner, 2007).

Whether stress acts positively or negatively on sexual reproduction might depend on its severity. Moderate light stress and the subsequent overproduction of reactive oxygen species (ROS) may enhance meiosis as a DNA repair mechanism (Hörandl and Hadacek, 2013). Indeed, prolonged photoperiods resulted in increased production of sexual ovules as shown for the facultatively apomictic *Ranunculus auricomus* (Klatt *et al.*, 2016) and *Paspalum cromyorrhizon* (Quarin, 1986). Severe cold stress, on the other hand, can disturb meiosis in several ways (De Storme and Geelen, 2014) and may result in abnormal or aborted meiotic products. When sexual reproduction is likely to fail, a change to the alternative apomeiotic pathway could increase the chance of successful reproduction in facultative apomictic plants.

In diploid *R. kuepferi*, cold stress could be severe enough to cause failure or delay of meiosis and sporogenesis, and concomitantly to trigger apomictic development in some of the ovules. Strikingly, in *R. kuepferi*, apomeiosis is coupled immediately to parthenogenesis, resulting in fully functional apomictic seed in 14 cases, while only four seeds were formed as B_III_ hybrids (Table 2). Tetraploids are probably more tolerant to cold stress, so that our treatments did not reveal a significant effect on proportions of apomictic seed formation. An indirect effect of polyploidy on buffering stress situations was postulated earlier (Hörandl and Hadacek, 2013), but requires further study.

Moreover, despite regular watering and identical light regimes in our temperature treatments, any influence on plant physiology by, for example, temporary soil drought after frost nights or general light stress due to the artificial light conditions cannot be ruled out. Stress response in plants is quite complex, and the combination of several stress conditions can, for example, initiate opposing signalling pathways (Suzuki *et al.*, 2014). This might have affected our diploid outdoor group in particular, as these showed significantly higher flowering and fruiting rates than all other groups. For simulating alpine conditions, however, it remains to be tested if longer frost treatments (>8 h) or colder temperatures (lower than –1 °C) would increase the proportions of apomictic seeds.

In addition to the observed reproductive flexibility of the diploid and tetraploid *R. kuepferi* cytotype in our temperature experiment and at natural sites in the Alps (Schinkel *et al.*, 2016), we also found hints for phenotypic plasticity in flower development and mode of reproduction. Some individuals of *R. kuepferi* did not flower every year. We recorded individuals with flowers in two or three successive years but also plants that flowered just every 2 years. All individuals seem to be capable of producing apomictic seeds. Also partial apomixis with unreduced apomeiotic female gametes fertilized by reduced pollen occurred in our experimental warm treatments as well as on natural sites (Schinkel *et al*., 2016, 2017). Considering the small number of individuals that could be evaluated, and the limited number of seeds available from a single plant, results on phenotypic plasticity of apomixis should be treated with caution. Apomixis is a heritable trait, but whether individuals reproduce facultatively or obligatorily sexually/asexually is not fixed in *R. kuepferi* but can vary in different growing seasons. One possible explanation is that expression of apomixis is under epigenetic control, as previously suggested by Grimanelli (2012). Environmental stress can change methylation patterns and transposon activity, which can be passed on to the offspring of asexual plants (Verhoeven *et al.*, 2010; Verhoeven and Preite, 2014).

As apomictic reproduction was predominantly reported for polyploid plants (Carman, 1997) and was also known for the tetraploid *R. kuepferi* cytotype, it was assumed previously that a polyploidization event took place before apomixis developed (Cosendai and Hörandl, 2010). This hypothesis must be rejected for *R. kuepferi* because we found full functional apomixis (i.e. apomeiosis coupled to parthenogenesis and endosperm development) in some individuals of the diploid cytotype in this experiment as well as in the wild (Schinkel *et al*., 2016, 2017). However, apomictic seeds were formed only in very low frequencies in diploid *R. kuepferi*, and natural diploid apomixis is also rare in other plants (Shah *et al.*, 2016).

Our findings contradict earlier hypotheses that polyploidy would be the major factor for the induction of apomixis (Carman, 1997; Koltunow and Grossniklaus, 2003). In contrast, our data suggest that diploid plants do have an inherent, latent potential for apomictic seed formation. Apomeiotic initials also occur spontaneously in other species, but usually infrequently and without coupling to parthenogenesis (Noyes, 2007; Ortiz *et al.*, 2013). The potential for apomeiotic, unreduced embryo sac formation is in general probably activated by a disturbance of meiosis and megasporogenesis. This disturbance could, on the one hand, be a consequence of interspecific hybridization, as assumed for diploid *Boechera* (Sharbel *et al.*, 2010) and for diploid *Ranunculus auricomus* primary hybrids (Hojsgaard *et al.*, 2014*b*). However, for diploid *R. kuepferi*, a hybrid origin is unlikely (Cosendai *et al.*, 2013). In this originally warm-adapted species, disturbances of meiosis and sporogenesis could have emerged when the species experienced colder climatic conditions during post-glacial re-colonization of higher regions in the Alps (Kirchheimer *et al.*, 2016). However, cold-induced apomeiosis and the successful coupling to parthenogenesis probably do not happen in diploids frequently enough to replace the established sexual pathway.

B_III_ seed formation could have been the first step towards polyploidization. Tetraploidy could arise in one step from diploid plants when an unreduced egg cell is fertilized by unreduced pollen. The formation of unreduced gametes is usually a rare event in a diploid population, but certain individuals could do so more frequently (Sora *et al.*, 2016) and thereby directly form a new polyploid cytotype. The more likely way to tetraploidy, however, is via a triploid bridge (Bretagnolle and Thompson, 1995) built by triploid offspring that resulted from the fusion of unreduced and reduced gametes. In a second step, an unreduced gamete from the triploid can be fertilized by a reduced gamete and form a tetraploid embryo. Seeds with unreduced but fertilized egg cells (female B_III_ hybrids) produced by our warm-treated *R. kuepferi* plants support this scenario (Table 2). The endosperm ploidy levels of these B_III_ hybrid seeds revealed the contribution of reduced male gametes only. We assume a ‘female triploid bridge’ to be the step between the diploid and tetraploid *R. kuepferi* cytotype as B_III_ hybrids from wild plants are also predominantly derived from the fertilization of unreduced egg cells with reduced male gametes (Schinkel *et al*., 2016, 2017). Facultatively apomictic triploids as a bridge between sexual diploids and predominantly apomictic tetraploids were also considered for *Amelanchier* (Rosaceae) (Burgess *et al.*, 2014). Altogether, frequencies of B_III_ formation were too low in our experiments to detect significant differences between warm and cold treatments (Fig. 3). However, our experiments confirm the finding of a large-scale screening on wild populations by Schinkel *et al*. (2016, 2017) that polyploidization happens mostly via unreduced female gametes. The role of polyploidy for the quantitative establishment of apomixis remains unclear. On the one hand, the remaining sexual pathway in these autopolyploids might be disturbed by multivalent formation at meiosis and unequal segregation of chromosomes in megaspores (Cosendai *et al.*, 2011). Low temperatures may further exacerbate these problems by disturbing spindle and cell wall formation during meiosis, as is known for male development (De Storme and Geelen, 2014; Hörandl and Mirzaghaderi, 2016). Hence, initial low frequencies of apomictic seed formation in tetraploids may have increased indirectly upon failure of the sexual pathway, especially at higher altitudes and under colder conditions in the Alps (Kirchheimer *et al.*, 2016; Schinkel *et al.*, 2016). Selection for apomictic seeds may have stabilized the above-mentioned epigenetic control mechanisms over generations. Thus, polyploidy is probably not directly the functional trigger for apomixis, but rather helps to establish apomixis indirectly as the predominant (but not necessarily obligate) mode of reproduction of the tetraploid cytotype.

### Conclusions and perspectives

In temperature experiments and at natural sites (Schinkel *et al.*, 2016), proportions of sexual vs. apomictic seeds varied in diploids and tetraploids, and depended on the plant seed set, indicating that both *R. kuepferi* cytotypes can use apomictic reproduction as a flexible strategy. The diploid cytotype was expected to be warm adapted, while the tetraploid cytotype was thought to be better adapted to colder environments via late flowering, overinvestment in carpel and ovule production, and apomixis. Indeed, flowering and fruiting rates were only higher in diploids under lowland outdoor conditions, while tetraploids were not significantly affected by the treatment conditions. Nevertheless, the seed production was significantly negatively affected by cold temperatures and frost in both cytotypes. Early-flowering frost-sensitive diploids were significantly negatively affected by the applied cold temperatures and frost shocks regarding seed set and reproduction mode. A change to more apomictic seed formation after cold treatments was observed for diploids, while tetraploids remained unaffected. However, single individuals expressed phenotypic plasticity in their mode of reproduction in consecutive years, suggesting an influence of epigenetic control mechanisms for expression of reproductive phenotypes. Further experiments with frost shocks at different stages of the plants life cycle [e.g. (1) end of the last growth period during putative initiation of buds and (2) start of the growth period when meiosis proceeds], and extensive microscopic analyses of treated (young) buds would be helpful to investigate further the temperature effect on female gamete formation in *R. kuepferi*. Rare events of partial apomixis in diploids can result in polyploidization, and triploid progeny could represent a bridge to the emergence of the tetraploid cytotype. In contrast to the general assumption that polyploidy developed prior to apomixis, in *R. kuepferi* these processes might have evolved in reverse order. Possibly, warm-adapted lowland diploids experienced colder conditions during post-glacial colonization of higher elevations, and this experience could have triggered apomixis and polyploidization. Successively, apomixis and a cold-tolerant phenology were established in the tetraploid cytotype at higher altitudes of the Alps.

## SUPPLEMENTARY DATA

Supplementary data are available online at https://academic.oup.com/aob and consist of the following. Table S1: *Ranunculus kuepferi* populations in the experimental study and the collection sites in the European Alps. Table S2: FCSS data for embryo and endosperm ploidy in single seeds produced by diploid and tetraploid *Ranunculus kuepferi* in experiments with special cases of underlying reproduction modes. Table S3: statistical characteristics of the effects of temperature treatments on the reproductive fitness (production of well-developed seeds) in diploid and tetraploid *Ranunculus kuepferi* plants. Table S4: statistical characteristics of the effects of temperature treatments on the production of sexual, apomictic and partial apomictic (B_III_) seeds by diploid and tetraploid *Ranunculus kuepferi* plants. Figure S1: temperature measured at ground level of *Ranunculus kuepferi* pots placed outdoors in the Botanical Garden of the University Göttingen during the experimental period in 2014. Figure S2: representative flow cytometry histograms for the six modes of reproduction. Figure S3: influence of the temperature treatment on the mode of reproduction in diploid and tetraploid *Ranunculus kuepferi* plants in the first and the second experimental year.

Supplementary-Tables_fig
