# Supplementary material for: Effects of cold treatments on fitness and mode of reproduction in the diploid and polyploid alpine plant Ranunculus kuepferi (Ranunculaceae)
Source: Ann Bot. 2018 Feb 15;121(7):1287–98. doi: 10.1093/aob/mcy017 (PMC6007502; doi:10.1093/aob/mcy017)
Supplement: Supplementary-Tables_fig [file mcy017_suppl_supplementary-tables_fig.doc]

Supplementary Tables S1-S4; Supplementary Fig. S1-S3

Table S1. *Ranunculus kuepferi* populations in the experimental study and the collection sites in the European Alps. Pop = population number; Ploidy = Ploidy level of the individual plants used for the experiments; A = Austria, CH = Switzerland, F = France, I = Italy, North and East denote coordinates; Altitude in m a.s.l. (according to Schinkel *et al.*, 2016).

| **Pop** | **Ploidy** | **Country** | **Region** | **North** | **East** | **Altitude** |
| --- | --- | --- | --- | --- | --- | --- |
| 3 | 2x | F | Provence-Alpes-Côte d'Azur | 44.20028 | 7.15639 | 2291 |
| 16 | 4x | F | Provence-Alpes-Côte d'Azur | 45.03483 | 6.40242 | 2078 |
| 17 | 4x | F | Provence-Alpes-Côte d'Azur | 45.05069 | 6.39078 | 2357 |
| 23 | 2x | F | Provence-Alpes-Côte d'Azur | 43.74931 | 6.65744 | 1616 |
| 24 | 2x | F | Provence-Alpes-Côte d'Azur | 44.15083 | 6.54361 | 1925 |
| 25 | 2x | F | Provence-Alpes-Côte d'Azur | 44.90128 | 5.47617 | 1435 |
| 26 | 2x | F | Provence-Alpes-Côte d'Azur | 44.90033 | 5.46900 | 1456 |
| 27 | 2x | F | Rhônes-Alpes | 44.83933 | 5.42422 | 1449 |
| 28 | 2x | I | Piemonte | 44.24414 | 7.62906 | 1685 |
| 29 | 2x | I | Piemonte | 44.23111 | 7.61722 | 2020 |
| 30 | 2x | I | Piemonte | 44.18458 | 7.62700 | 1743 |
| 31 | 2x | I | Piemonte | 44.19522 | 7.65922 | 1937 |
| 32 | 2x | I | Piemonte | 44.20667 | 7.14750 | 2320 |
| 33 | 2x | I | Piemonte | 44.21305 | 7.14667 | 2328 |
| 34 | 4x | F | Rhônes-Alpes | 45.24244 | 6.95189 | 2120 |
| 36 | 4x | F | Rhônes-Alpes | 45.38542 | 7.04372 | 2152 |
| 37 | 4x | I | Val d'Aosta | 45.61622 | 7.55261 | 2115 |
| 38 | 4x | F | Rhônes-Alpes | 45.67822 | 6.87800 | 2182 |
| 40 | 4x | CH | Valais | 46.08250 | 7.01250 | 1860 |
| 41 | 4x | I | Val d'Aosta | 45.93195 | 7.63944 | 2174 |
| 42 | 4x | CH | Valais | 46.43300 | 7.86492 | 1789 |
| 43 | 4x | CH | Valais | 46.25086 | 8.01800 | 2012 |
| 45 | 4x | CH | Valais | 46.57119 | 8.41056 | 2400 |
| 46 | 4x | CH | Tessin | 46.56367 | 8.79842 | 1937 |
| 47 | 4x | CH | Graubunden | 46.54628 | 9.21139 | 2211 |
| 48 | 4x | CH | Graubunden | 46.47153 | 9.72889 | 2262 |
| 50 | 4x | CH | Graubunden | 46.41275 | 10.02408 | 2322 |
| 51 | 4x | A | Tyrol | 47.14756 | 10.24817 | 2286 |
| 53 | 4x | CH | Graubunden | 46.54856 | 10.43431 | 2456 |
| 54 | 4x | I | Lombardia | 46.27239 | 10.57506 | 2303 |
| 55 | 4x | A | Tyrol | 46.87197 | 10.70836 | 2557 |
| 58 | 4x | I | Trentino Alto Adige/ Südtirol | 46.45667 | 11.88814 | 2117 |
| 66 | 4x | I | Trentino Alto Adige/ Südtirol | 46.95562 | 11.51737 | 2101 |
| 74 | 4x | A | Osttirol | 46.69904 | 12.44026 | 2117 |
| 75 | 4x | CH | Graubunden | 46.52845 | 9.81119 | 2678 |
| 78 | 4x | CH | Valais | 46.22506 | 7.15989 | 2000 |
| 79 | 4x | CH | Graubunden | 46.98753 | 10.35919 | 2280 |
| 82 | 4x | I | Lombardia | 46.53867 | 10.43525 | 2500 |
| 83 | 4x | A | Tyrol | 47.04070 | 12.69105 | 2271 |
| 84 | 4x | A | Carinthia | 47.06989 | 12.84618 | 2236 |
| 85 | 4x | A | Carinthia | 47.05495 | 12.86219 | 2184 |
| 88 | 4x | CH | Graubunden | 46.51597 | 9.82125 | 2300 |
| 89 | 4x | CH | Graubunden | 46.65067 | 9.75561 | 2265 |
| 90 | 4x | CH | Valais | 46.12433 | 7.51753 | 2477 |
| 92 | 4x | CH | Graubunden | 46.42364 | 9.63836 | 2260 |
| 93 | 4x | CH | Valais | 46.20853 | 7.72417 | 2405 |
| 96 | 4x | F | Provence-Alpes-Côte d'Azur | 44.81775 | 6.73211 | 2300 |
| 103 | 4x | I | Lombardia | 46.49211 | 10.20758 | 2290 |
| 106 | 4x | I | Trentino Alto Adige/ Südtirol | 46.94659 | 11.83144 | 2142 |
| 108 | 4x | CH | Graubunden | 46.45392 | 9.98694 | 2171 |
| 110 | 4x | I | Trentino Alto Adige/ Südtirol | 46.99229 | 12.11355 | 2312 |
| 111 | 4x | F | Provence-Alpes-Côte d'Azur | 44.27900 | 6.71922 | 2243 |
| 112 | 2x | F | Provence-Alpes-Côte d'Azur | 43.85250 | 6.35278 | 1626 |
| 115 | 2x | F | Provence-Alpes-Côte d'Azur | 44.24500 | 6.75611 | 1891 |
| 116 | 2x/4x | F | Provence-Alpes-Côte d'Azur | 44.24797 | 6.76194 | 1953 |
| 117 | 2x | F | Provence-Alpes-Côte d'Azur | 43.74556 | 6.65583 | 1632 |
| 118 | 2x | I | Piemonte | 44.23161 | 7.62833 | 1636 |
| 119 | 2x | I | Piemonte | 44.26778 | 7.21111 | 1820 |
| 120 | 2x | I | Piemonte | 44.22333 | 7.12058 | 1966 |
| 121 | 4x | F | Provence-Alpes-Côte d'Azur | 44.20639 | 7.11556 | 1710 |
| 202 | 2x | F | Provence-Alpes-Côte d'Azur | 44.15900 | 6.71461 | 1829 |
| 203 | 2x | F | Provence-Alpes-Côte d'Azur | 44.16294 | 6.71081 | 1840 |
| 208 | 4x | F | Provence-Alpes-Côte d'Azur | 44.30244 | 6.56097 | 1924 |
| 233 | 2x | F | Provence-Alpes-Côte d'Azur | 44.12946 | 6.96878 | 2185 |

Table S2. FCSS data for embryo and endosperm ploidy in single seeds produced by diploid and tetraploid *Ranunculus kuepferi* in experiments (2014 and 2015 combined) with special cases of underlying reproduction modes. Cx reflects ploidy based on DNA content, m, maternal genome contribution, p, paternal genome contribution. These data were excluded from further statistics.

| **Assumed reproduction mode** | **Genome contribution to Embryo / Endosperm** | **Embryo : Endosperm** | **Peak Index** | **Number of observations (seeds, ssFCSS) in two experimental years** | | | |
| --- | --- | --- | --- | --- | --- | --- | --- |
| **Diploid Plants** | Egg cell + sperm nucleus / fused polar nuclei + sperm nucleus (nuclei) |  |  | Cold | Warm | Outdoor^a^ | Total |
| Sexual, with unbalanced meiosis | <1Cx(m)+<1Cx(p) / 2Cx(m)+1Cx(p) | 1.75:3 | 1.7-1.8 | 10 | 0 | 0 | 10 |
| Sexual, with unbalanced meiosis | <1Cx(m)+<1Cx(p) / 2Cx(m)+1Cx(p) | 1.5:3 | 2.0 | 2 | 0 | 0 | 2 |
| Apospory with polyspermy | 2Cx(m)+1Cx(p) / 4Cx(m)+3Cx(p) | 3:7 | 2.3 | 0 | 4 | 0 | 4 |
| Apospory with polyspermy | 2Cx(m)+1Cx(p) / 4Cx(m)+4Cx(p) | 3:8 | ~2.6 | 0 | 5 | 0 | 5 |
| Apospory with polyspermy | 2Cx(m)+2Cx(p) / 4Cx(m)+5Cx(p) | 4:9 | 2.3 | 0 | 7 | 0 | 7 |
| Apospory with endosperm endopolyploidy | 2Cxm+2Cx(p) / (4Cx(m)+2Cx(p))*2 | 4:12 | 3.0 | 0 | 1 | 0 | 1 |
| **Tetraploid Plants** |  |  |  |  |  |  |  |
| Dihaploid | 2Cx(m)+0Cx(p) / ? | 2:3 | 1.5 | 0 | 1 | 0 | 1 |
| Dihaploid with autonomous endosperm | 2Cx(m)+0Cx(p) / 4Cx(m)+0Cx(p) | 2:4 | 2.0 | 0 | 0 | 1 | 1 |
| Dihaploid with pseudogamous endosperm | 2Cx(m)+0Cx(p) / 4Cx(m)+2Cx(p) | 2:6 | 3.0 | 0 | 0 | 2 | 2 |
| Hypotetraploid aneuploidy with autonomous endosperm | 3Cx(m)+0Cx(p) / 6Cx(m)+0Cx(p) | 3:6 | 2.0 | 1 | 0 | 0 | 1 |
| Hypotetraploid aneuploidy with pseudogamous endosperm | 3Cx(m)+0Cx(p) / 6Cx(m)+4Cx(p) | 3:10 | 3.3 | 1 | 0 | 0 | 1 |
| Apospory with endopolyploidy | 4Cx(m)+0Cx(p) / (8Cx(m))*2+2Cx(p) | 4:18 | 1:4.5 | 0 | 1 | 0 | 1 |
| Hypertetraploid aneuploidy with autonomous endosperm | 5Cx(m)+0Cx(p) / 10Cx(m)+0Cx(p) | 5:10 | 2.0 | 0 | 1 | 0 | 1 |
| B_III_ hybrid with polyspermy | 4Cx(m)+2Cx(p) / 8Cx(m)+6Cx(p)^b^ | 6:14 | 2.3 | 0 | 1 | 0 | 1 |

^a^: outdoor group in 2014 only, plants in Göttingen Old Botanical Garden

^b^: or trinucleate endosperm 12Cx(m)+2Cx(p), see Schinkel et al. 2016

Table S3. Statistical characteristics of the effects of temperature treatments on the reproductive fitness (proportion of well-developed seeds) in diploid and tetraploid *Ranunculus kuepferi* plants. P-values in bold indicate significant differences between temperature treatments.

|  | **2014** | | | | | |
| --- | --- | --- | --- | --- | --- | --- |
|  | Diploid plants | | | Tetraploid plants | | |
|  | Cold | Warm | Outdoor | Cold | Warm | Outdoor |
|  | **Proportion of well developed seeds (%)** | | | | | |
| Median | 50.00 | 82.70 | 77.10 | 0.40 | 27.00 | 20.40 |
| Mean | 47.21 | 72.72 | 72.02 | 6.12 | 30.44 | 22.24 |
| SD | 25.15 | 28.76 | 23.13 | 12.15 | 18.04 | 17.72 |
| *N* | 21 | 18 | 34 | 20 | 13 | 13 |
| *p*-value^1^ | **0.002** | **0.002** |  | **0.000** | **0.000** |  |
|  | **0.001** |  | **0.001** | **0.006** |  | **0.006** |
|  |  | 0.637 | 0.637 |  | 0.182 | 0.182 |
|  | **2015** | | | | | |
|  | Diploid plants | | | Tetraploid plants | | |
|  | Cold | Warm |  | Cold | Warm |  |
|  | **Proportion of well developed seeds (%)** | | | | | |
| Median | 0.00 | 54.17 |  | 0.19 | 6.36 |  |
| Mean | 4.99 | 46.04 |  | 3.54 | 11.53 |  |
| SD | 9.26 | 29.09 |  | 11.09 | 11.99 |  |
| *N* | 56 | 65 |  | 75 | 61 |  |
| *p*-value^1^ | **0.000** | |  | **0.000** | |  |

*N*: number of individuals

^1^: level of significance, pairwise Mann-Whitney U-Tests between temperature treatments of the same ploidy level with α = 0.05 and arcsine transformed data

Table S4. Statistical characteristics of the effects of temperature treatments on the production of sexual, apomictic, and partial apomictic (B_III_) seeds by diploid and tetraploid *Ranunculus kuepferi* plants. *P*-values in bold indicate significant differences between temperature treatments.

| (a) both years combined | | | | | | | | | | |  |
| --- | --- | --- | --- | --- | --- | --- | --- | --- | --- | --- | --- |
|  | | Diploid plants | | | | | Tetraploid plants | | | |  |
| Treatment | | Cold | | Warm | | | Cold | | Warm | |  |
| *N* | | 32 | | 74 | | | 37 | | 58 | |  |
|  | | **Proportion of sexual seeds (%)** | | | | | | | | |  |
| Median | | 100.00 | | 100.00 | | | 0.00 | | 0.00 | |  |
| Mean | | 97.16 | | 96.70 | | | 4.57 | | 6.90 | |  |
| SD | | 5.67 | | 16.43 | | | 11.67 | | 15.96 | |  |
| *p*-value^1^ | | **0.050** | | | | | 0.267 | | | |  |
|  | | **Proportion of apomictic seeds (%)** | | | | | | | | |  |
| Median | | 0.00 | | 0.00 | | | 100.00 | | 100.00 | |  |
| Mean | | 2.84 | | 0.60 | | | 95.43 | | 92.33 | |  |
| SD | | 5.67 | | 2.56 | | | 11.67 | | 16.73 | |  |
| *p*-value^1^ | | **0.009** | | | | | 0.158 | | | |  |
|  | | **Proportion of B_III_ hybrids (partial apomixis) (%)** | | | | | | | | |  |
| Median | | 0.00 | | 0.00 | | | 0.00 | | 0.00 | |  |
| Mean | | 0.00 | | 2.70 | | | 0.00 | | 0.78 | |  |
| SD | | 0.00 | | 16.33 | | | 0.00 | | 3.72 | |  |
| *p*-value | | 0.350 | | | | | 0.162 | | | |  |
|  | (b) individual years | | | | | | | | | | |
|  | **2014** | | | | | | | | | | |
|  | Diploid plants | | | | | Tetraploid plants | | | | | |
| Treatment | Cold | | Warm | | Outdoor | Cold | | Warm | | Outdoor | |
| *N* | 18 | | 18 | | 33 | 7 | | 13 | | 11 | |
|  | **Proportion of sexual seeds (%)** | | | | | | | | | | |
| Median | 100.00 | | 100.00 | | 100.00 | 0.00 | | 0.00 | | 0.00 | |
| Mean | 96.06 | | 98.89 | | 99.09 | 2.86 | | 4.42 | | 15.66 | |
| SD | 5.82 | | 3.23 | | 2.92 | 7.56 | | 7.78 | | 32.09 | |
| *p*-value^1^ | 0.082 | | 0.082 | |  | 0.498 | | 0.498 | |  | |
|  | **0.021** | |  | | **0.021** | 0.419 | |  | | 0.419 | |
|  |  | | 0.818 | | 0.818 |  | | 0.857 | | 0.857 | |
|  | **Proportion of apomictic seeds (%)** | | | | | | | | | | |
| Median | 0.00 | | 0.00 | | 0.00 | 100.00 | | 100.00 | | 100.00 | |
| Mean | 3.93 | | 1.11 | | 0.91 | 97.14 | | 92.12 | | 83.43 | |
| SD | 5.82 | | 3.23 | | 2.92 | 7.56 | | 13.69 | | 31.74 | |
| p-value^1^ | 0.082 | | 0.082 | |  | 0.243 | | 0.243 | |  | |
|  | **0.021** | |  | | **0.021** | 0.276 | |  | | 0.276 | |
|  |  | | 0.818 | | 0.818 |  | | 0.974 | | 0.974 | |
|  | **Proportion of B_III_ hybrids (partial apomixis) (%)** | | | | | | | | | | |
| Median | 0.00 | | 0.00 | | 0.00 | 0.00 | | 0.00 | | 0.00 | |
| Mean | 0.00 | | 0.00 | | 0.00 | 0.00 | | 3.46 | | 0.91 | |
| SD | 0.00 | | 0.00 | | 0.00 | 0.00 | | 7.47 | | 3.02 | |
| *p*-value^1^ | 1.000 | | 1.000 | |  | 0.180 | | 0.180 | |  | |
|  | 1.000 | |  | | 1.000 | 0.425 | |  | | 0.425 | |
|  |  | | 1.000 | | 1.000 |  | | 0.348 | | 0.348 | |
|  | **Proportion of nonsexual seeds (apomictic and B_III_) (%)** | | | | | | | | | | |
| Median | 0.00 | | 0.00 | | 0.00 | 100.00 | | 100.00 | | 100.00 | |
| Mean | 3.93 | | 1.11 | | 0.91 | 97.14 | | 95.57 | | 84.34 | |
| SD | 5.82 | | 3.23 | | 2.92 | 7.56 | | 7.78 | | 32.09 | |
| *p*-value^1^ | 0.082 | | 0.082 | |  | 0.498 | | 0.498 | |  | |
|  | **0.021** | |  | | **0.021** | 0.419 | |  | | 0.419 | |
|  |  | | 0.818 | | 0.818 |  | | 0.857 | | 0.857 | |

|  | **2015** | | | | | |
| --- | --- | --- | --- | --- | --- | --- |
|  | Diploid plants | |  | Tetraploid plants | |  |
| Treatment | Cold | Warm |  | Cold | Warm |  |
| *N* | 14 | 56 |  | 30 | 45 |  |
|  | **Proportion of sexual seeds (%)** | | | | | |
| Median | 100.00 | 100.00 |  | 0.00 | 0.00 |  |
| Mean | 98.57 | 95.99 |  | 4.97 | 7.61 |  |
| SD | 5.34 | 18.78 |  | 12.51 | 17.64 |  |
| *p*-value^1^ | 1.000 | |  | 0.372 | |  |
|  | **Proportion of apomictic seeds (%)** | | | | | |
| Median | 0.00 | 0.00 |  | 100.00 | 100.00 |  |
| Mean | 1.43 | 0.43 |  | 95.03 | 92.39 |  |
| SD | 5.35 | 2.31 |  | 12.51 | 17.64 |  |
| *p*-value^1^ | 0.530 | |  | 0.372 | |  |
|  | **Proportion of B_III_ hybrids (partial apomixis) (%)** | | | | | |
| Median | 0.00 | 0.00 |  | 0.00 | 0.00 |  |
| Mean | 0.00 | 3.57 |  | 0.00 | 0.00 |  |
| SD | 0.00 | 18.73 |  | 0.00 | 0.00 |  |
| *p*-value^1^ | 0.476 | |  | 1.000 | |  |
|  | **Proportion of nonsexual seeds (apomictic and B_III_) (%)** | | | | | |
| Median | 0.00 | 0.00 |  | 100.00 | 100.00 |  |
| Mean | 1.43 | 4.01 |  | 95.03 | 92.39 |  |
| SD | 5.35 | 18.78 |  | 12.51 | 17.64 |  |
| *p*-value^1^ | 1.000 | |  | 0.372 | |  |

*N*: number of individuals

^1^: level of significance, pairwise Mann-Whitney U-Tests between temperature treatments of the same ploidy level with α = 0.05 and arcsine transformed data


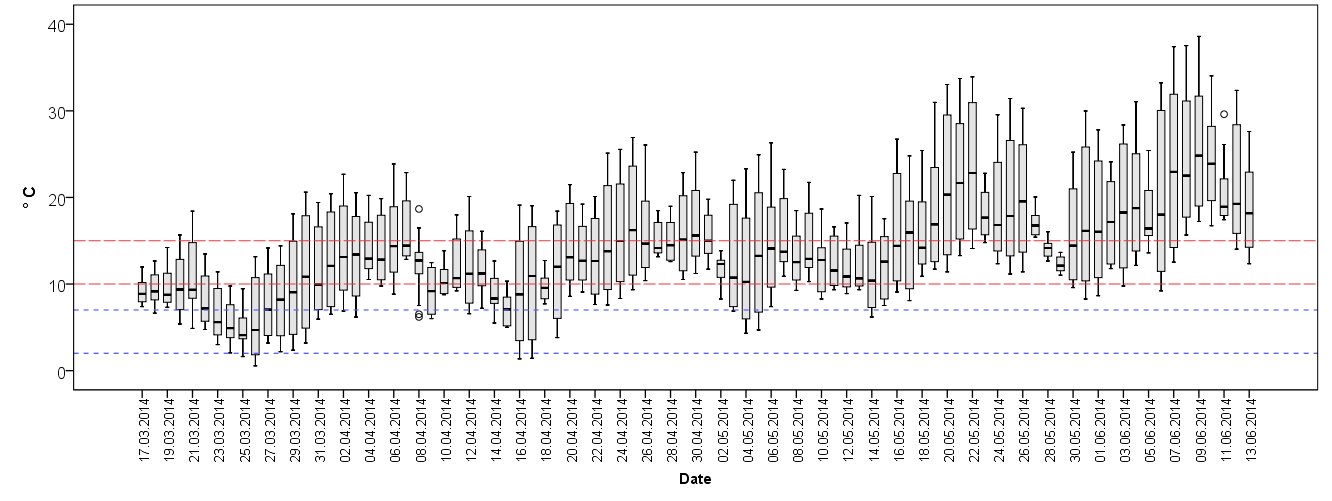


Fig. S1 Temperature (°C) measured at ground level of *Ranunculus kuepferi* pots placed outdoor in the Old Botanical Garden of the University Göttingen during the experimental period in 2014. Boxplots represent all measurements per day (hourly) and horizontal lines in boxplots indicate the median temperature. The upper two dashed lines mark the day/night temperatures in the warm treatment (+15/+10°C), the lower two dashed lines mark the day/night temperatures in the cold treatment (+7/+2°C, periodic frost application not indicated). Temperatures were logged with iButton, Maxim Integrated Products, Sunyvale, CA, USA.


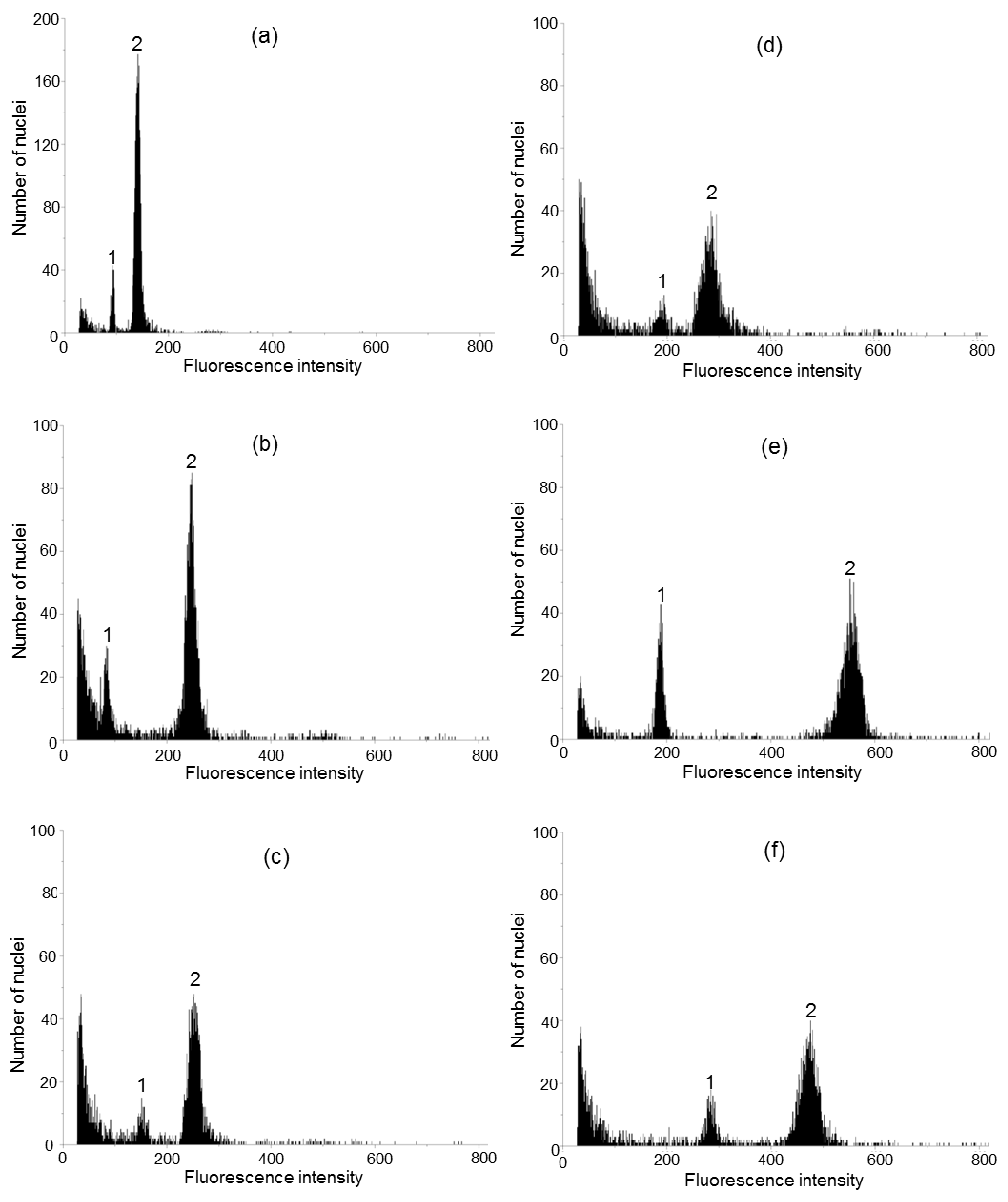


Fig. S2. Representative flow cytometry histograms for the six modes of reproduction: Histograms of a sexual seed (a), an asexual seed (b) and a B_III_-seed (c) produced by facultative apomictic diploid *Ranunculus kuepferi* plants and histograms of a sexual seed (d), an asexual seed (e) and a B_III_-seed (f) produced by facultative apomictic tetraploid *Ranunculus kuepferi* plants. The fluorescence intensity (relative indication) reflects the relative DNA content of the measured nuclei. Peak 1, nuclei of the embryo tissue; peak 2, nuclei of the endosperm tissue. Peak indices (embryo DNA content : endosperm DNA content) of 1 : 1.5 (a, d) and 1 : 3.0 (b, e) indicate a sexual seed and an asexual seed, respectively. Histograms of BIII-seeds show an embryo peak shift and ratio of 1 : 1.7.


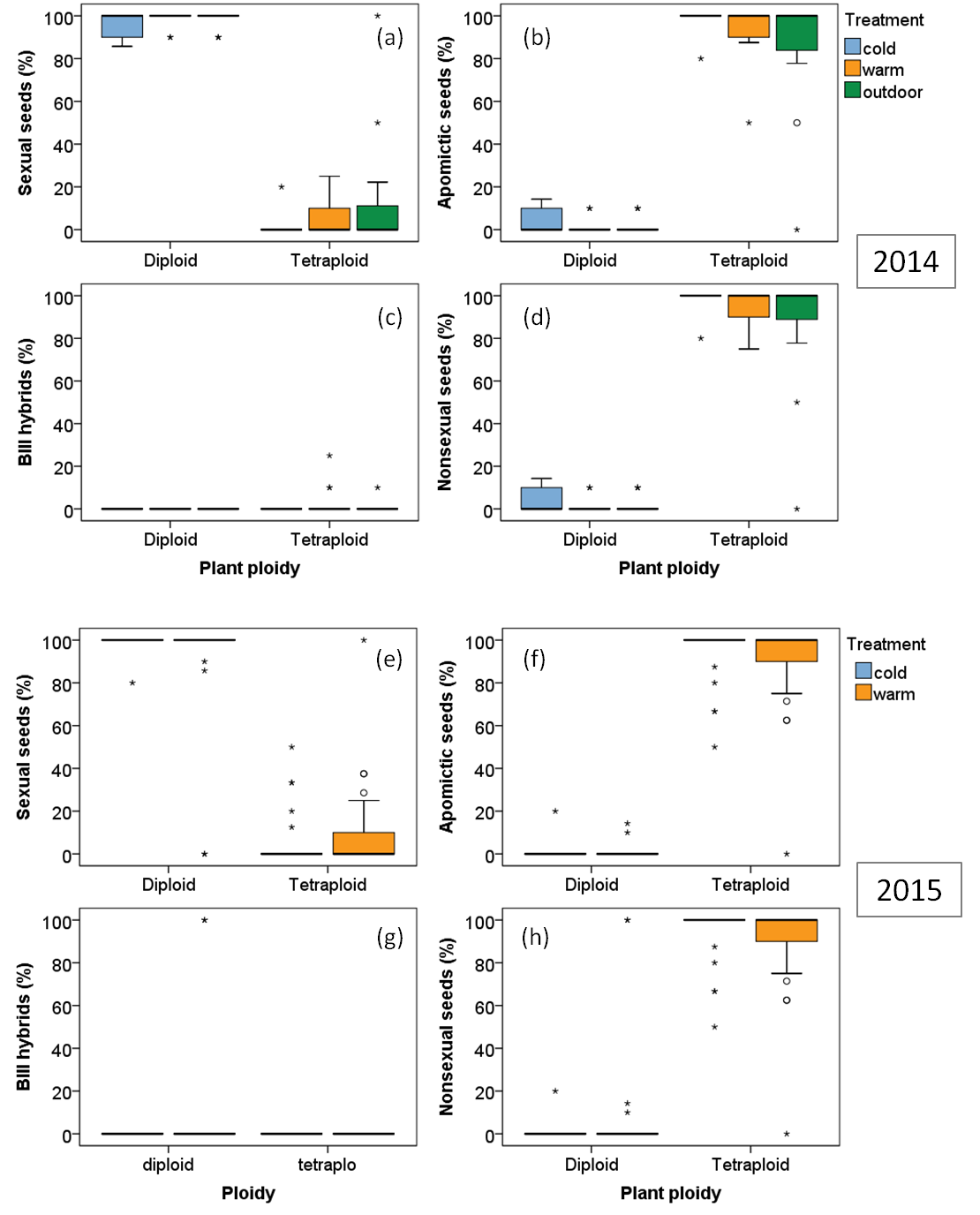


Fig. S3. Influence of the temperature treatment on the mode of reproduction in diploid and tetraploid *Ranunculus kuepferi* plants in the first experimental year 2014 (a – d) and the second experimental year 2015 (e – h). Boxplots show the percentages of sexual seeds (a) and (e), apomictic seeds (b) and (f), partially asexual seeds (B_III_ hybrids) (c) and (g), and nonsexual seeds (combines apomictic and partially asexual seeds) (d) and (h) for all individuals of the cold, warm and outdoor treatments. For test statistics see Table S4b.
